# Augmentation of Arthroscopic Rotator Cuff Repair Using Enthesis Interpositional Allograft and Minced Autologous Subacromial Bursa

**DOI:** 10.1002/atn2.70273

**Published:** 2026-09-30

**Authors:** Matthew D. Smith, Shyon K. Ahmadi, James M. Gregory

**Affiliations:** ^1^ Department of Orthopaedic Surgery University of Texas Health Science Center at Houston Houston Texas U.S.A.

## Abstract

Multiple strategies for rotator cuff augmentation have been developed to promote tendon healing and decrease retear rates. Previously published techniques and clinical studies have primarily emphasized biologic adjuncts intended to alter the local healing environment at the repair site or structural reinforcement with grafts. Although autologous subacromial bursal tissue has a potential benefit through local delivery of mesenchymal stem cells, it is difficult to maintain at the repair site. In vitro studies of scaffold augmentation with a demineralized interpositional graft are promising and would allow for the addition of biologic adjuncts directly at the repair site. Each strategy for augmentation offers distinct advantages and disadvantages, and the complimentary mechanisms of graft and biologic augmentation allow them to be combined. This technical note describes an augmentation technique for arthroscopic rotator cuff repair that incorporates an interpositional biphasic allograft in conjunction with autologous subacromial bursal tissue. The described technique provides a scaffold for healing at the enthesis while simultaneously delivering autologous tissue to promote tendon‐to‐bone healing. A combined strategy may represent a practical approach to optimizing repair integrity in patients at increased risk of failure after rotator cuff repair.

## Graphical abstract

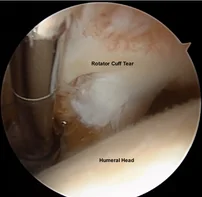

**VIDEO 1:** Augmentation of arthroscopic rotator cuff repair using a biphasic interpositional allograft and minced autologous subacromial bursa. Video content can be viewed at https://doi.org/10.1002/atn2.70273.

Retear rates after arthroscopic rotator cuff repair (RCR) are reported in up to 60% of cases.^1^, ^2^ Advances in augmentation strategies have been developed to improve tendon healing rates. Techniques for RCR augmentation include onlay grafts, interpositional (inlay) grafts, and biologic augmentation with platelet‐rich plasma, bone marrow aspirate concentrate, or minced autologous subacromial bursa.^3^, ^4^, ^5^ However, there is no gold standard, as investigations of different techniques show mixed results.

Several authors have described techniques for RCR augmented with a biphasic interpositional allograft.^5^, ^6^, ^7^ These techniques highlight an interpositional (inlay) graft that serves as a scaffold for rotator cuff healing at the bone‐tendon interface. Interpositional augmentation with a biphasic allograft has shown safety and promising results in animal studies.^8^, ^9^, ^10^ In addition to a scaffold, biologic augmentation can be incorporated to promote a cellular response for tendon healing. Randomized controlled trials evaluating biologic augmentation have shown mixed results. Gregory et al. found no difference in RCR healing rates with reimplantation of autologous subacromial bursa on the surface of the repair construct.^11^ However, Cole et al. showed lower retear rates with bone marrow aspirate concentrate when injected at the bone‐tendon junction and rotator cuff footprint.^12^

This technical note outlines RCR augmentation with both a biphasic interpositional allograft and minced autologous subacromial bursa. Our technique includes collection of subacromial tissue with a filter (GraftNet Autologous Tissue Collector; Arthrex, Naples, FL), followed by a double‐row suture repair with an interpositional enthesis graft (BioEnthesis; Sparta Biopharma, Madison, NJ) and insertion of the collected subacromial bursal tissue at the repair site. This technique provides structural augmentation at the enthesis, and the porous architecture of the graft helps retain subacromial bursal tissue at the bone‐tendon interface. The goal of our approach is to combine the benefits of a scaffold with modulation of the tendon healing response from local cell populations.

## SURGICAL TECHNIQUE

### Preoperative Assessment

A thorough history and physical examination with radiographs is performed. Advanced imaging is obtained in patients suspected to have a symptomatic rotator cuff tear. Patients with a rotator cuff tear on magnetic resonance imaging are counseled on operative versus nonoperative management. RCR with augmentation is indicated in patients at high risk of retear based on tear size, chronicity, muscle atrophy, patient age, greater tuberosity osteopenia, or other medical risk factors.

### Patient Positioning and Diagnostic Arthroscopy

All cases are performed under general anesthesia with an interscalene block. The patient is placed in the beach‐chair position. Diagnostic arthroscopy is performed using standard anterior and posterior portals. Intra‐articular assessment of the glenoid, humeral head, labrum, biceps tendon, and extent of rotator cuff tearing is documented. Once the rotator cuff tear is identified through the glenohumeral joint (Figure 1), the arthroscope is placed in the subacromial space. A lateral portal is created, and the subacromial bursa is visualized for an anterolateral and subdeltoid bursectomy.

**FIGURE 1:**
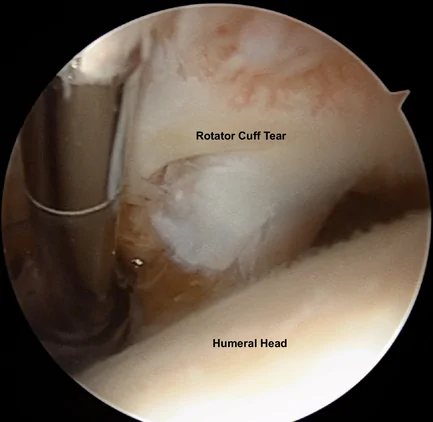
Right shoulder rotator cuff tear visualized through the glenohumeral joint from a posterior arthroscopic viewing portal with the patient in the beach‐chair position.

### Collation of Bursal Tissue, Footprint, and Graft Preparation

The suction collection device (GraftNet Autologous Tissue Collector; Arthrex) is attached to the shaver prior to bursectomy. The subacromial bursectomy is performed with a 4.5‐mm oscillating shaver (Figure 2), and the bursal tissue is collected. The rotator cuff tear is now better visualized in the subacromial space (Figure 3), allowing for assessment of tear dimensions, mobility, and repairability. The bursal tissue is extracted from the collection device and minced using a freer elevator and 11‐blade scalpel (Figure 4 and Video 1). Tears amenable to repair with enthesis graft augmentation are confirmed, and the biphasic interpositional allograft (BioEnthesis; Sparta Biopharma) is opened. The enthesis graft is soaked for a minimum of 15 minutes in arthroscopic fluid mixed with autologous minced subacromial bursa (Figure 5) prior to implantation. The rotator cuff footprint on the greater tuberosity of the humerus is decorticated with a shaver and arthroscopic burr (Figure 6). A ring curette may be used to remove any residual soft tissue from the greater tuberosity. The footprint is measured, and the enthesis graft is cut to the appropriate size (Figure 7A). Excess bursal tissue is collected and placed into a 3‐cc syringe (Figure 7B) for later reimplantation. The greater tuberosity is microfractured to stimulate bleeding (Figure 8).

**FIGURE 2:**
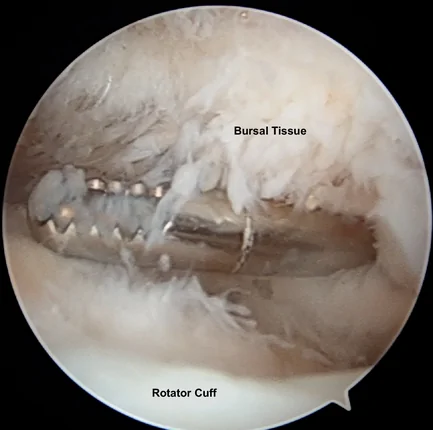
Right shoulder subacromial bursectomy performed with an arthroscopic shaver from a posterior arthroscopic viewing portal with the patient in the beach‐chair position.

**FIGURE 3:**
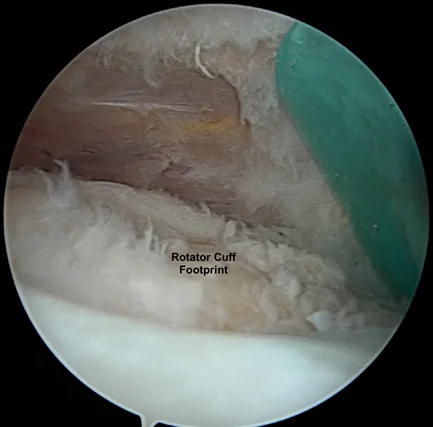
Right shoulder rotator cuff tear visualized through the subacromial space from a posterior arthroscopic viewing portal with the patient in the beach‐chair position.

**FIGURE 4:**
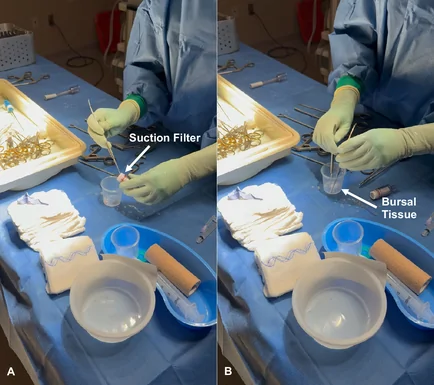
(A) Extraction of the collected subacromial bursa tissue from the suction filtration device and (B) preparation of the minced subacromial bursa tissue with a freer elevator and an 11‐blade scalpel.

**FIGURE 5:**
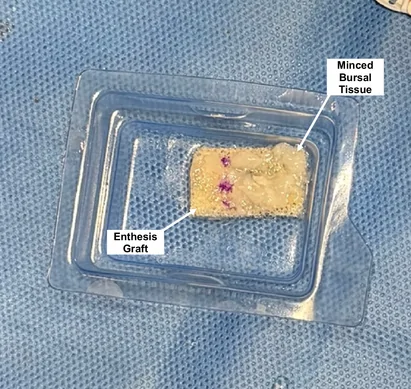
The enthesis graft is soaked in arthroscopic fluid mixed with autologous minced subacromial bursal tissue prior to implantation. The superficial side of the graft is marked for appropriate orientation.

**FIGURE 6:**
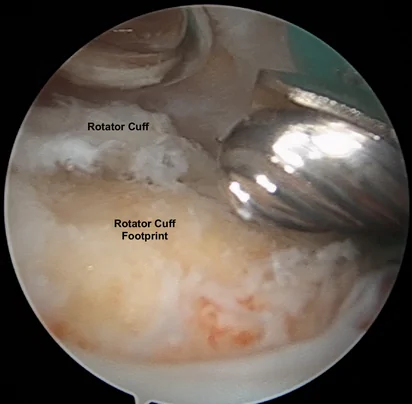
Decortication of the right shoulder rotator cuff footprint using an arthroscopic burr from a posterior arthroscopic viewing portal with the patient in the beach‐chair position.

**FIGURE 7:**
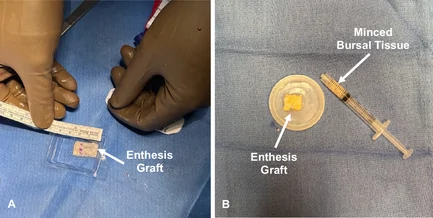
(A) The enthesis graft is measured and cut to the appropriate size of the prepared rotator cuff footprint. (B) The remainder of the subacromial bursa is placed into a 3‐cc syringe for later implantation after completion of rotator cuff repair.

**FIGURE 8:**
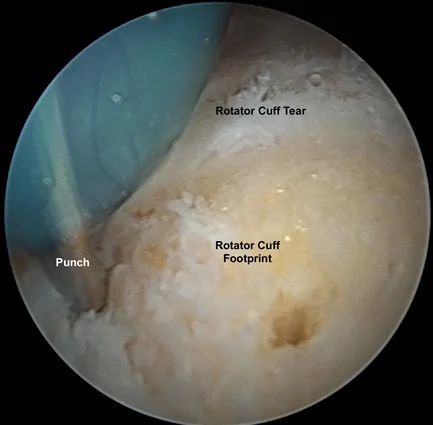
Right shoulder greater tuberosity is microfractured using an anchor punch and mallet from a lateral arthroscopic viewing portal with the patient in the beach‐chair position.

### Double‐Row Repair With Enthesis Graft

One to 3 anchors (2.3 mm Iconix Speed; Stryker, Kalamazoo, MI) are placed at the medial aspect of the footprint depending on the tear (Figure 9). Sutures (1.2 mm XBraid TT; Stryker) are passed through the rotator cuff tendon in a horizontal mattress fashion (Figure 10). A 12 × 40 mm silicon cannula (Gateway; Stryker) is modified to remove the dam (Figure 11A) and allow passage of the graft. The modified cannula is placed in the posterior portal (Figure 11B), and the enthesis graft is passed into the shoulder with an arthroscopic grasper or ringed forceps. The graft is then positioned at the decorticated rotator cuff footprint (Figure 12). The appropriate position and orientation of the graft between the rotator cuff footprint and undersurface of the tendon is critical. The medial sutures prevent the graft from displacing into the glenohumeral joint. The graft may be provisionally held in place with a spinal needle, or additional medial row sutures tensioned over the top of the graft and held through a lateral accessory portal. The rotator cuff tendon is then reduced over the enthesis graft, and sutures are passed in a crossing fashion through lateral knotless anchors (4.75 mm AlphaVent Knotless; Stryker) to create a transosseous equivalent double‐row repair. These sutures secure the enthesis graft in position between the tendon and bone (Figure 13). Temporary graft‐holding sutures are removed, and graft position is confirmed.

**FIGURE 9:**
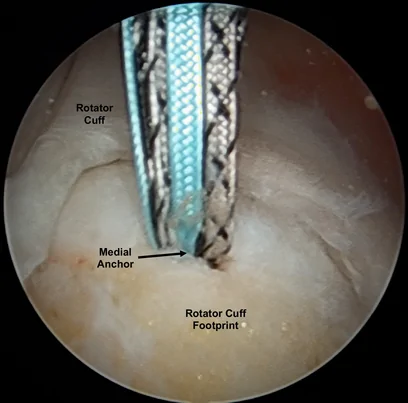
Right shoulder placement of the anchor at the medial aspect of the rotator cuff footprint from a lateral arthroscopic viewing portal with the patient in the beach‐chair position.

**FIGURE 10:**
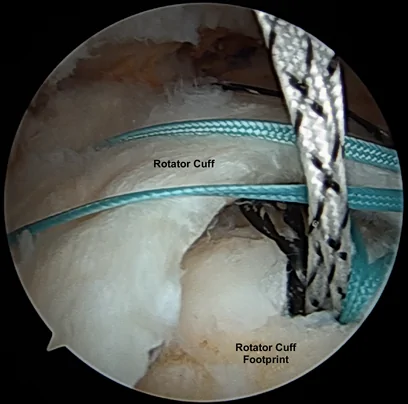
Suture is passed from medial anchors through the right shoulder rotator cuff tendon from a lateral arthroscopic viewing portal with the patient in the beach‐chair position.

**FIGURE 11:**
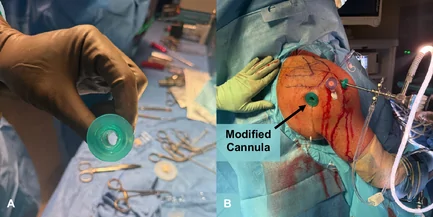
(A) A 12 × 40 mm silicon cannula with the dam removed to allow for passage of the enthesis graft. (B) Right shoulder in beach‐chair position with arthroscope in the lateral portal and modified cannula in the posterior arthroscopic portal.

**FIGURE 12:**
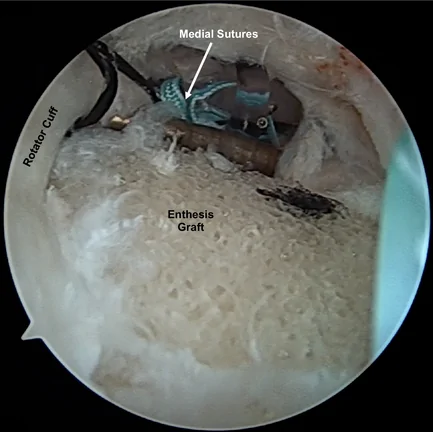
Positioning of the enthesis interpositional graft at the decorticated right shoulder rotator cuff footprint—lateral to the medial row sutures and deep to the rotator cuff tendon—viewing from a posterior arthroscopic portal with the patient in the beach‐chair position.

**FIGURE 13:**
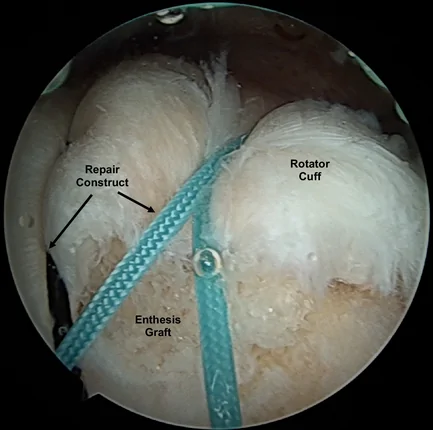
Completion of a right shoulder double‐row rotator cuff repair with enthesis interpositional graft after placement of 2 lateral row anchors, viewing from a lateral arthroscopic portal, with the patient in the beach‐chair position.

### Bursal Tissue Implantation

The shoulder is then evacuated of fluid. Once the shoulder is dry, the 3‐cc syringe containing isolated, minced subacromial bursal tissue is placed through a posterior portal and injected through the tendon, directly into the enthesis graft at the repair site (Figure 14). The arthroscope is removed, and standard portal closure is performed. Pearls and pitfalls of this technique are presented in Table 1.

**FIGURE 14:**
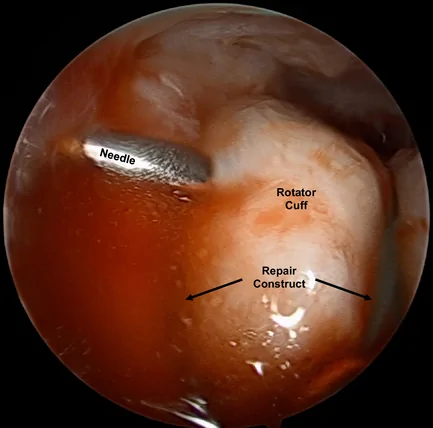
Injection of minced bursal tissue into the right shoulder rotator cuff tendon and enthesis graft after removal of arthroscopic fluid viewing from a lateral portal with the patient in the beach‐chair position.

**TABLE 1:** Pearls and Pitfalls for Arthroscopic RCR: A Biphasic Interpositional Allograft and Minced Autologous Subacromial Bursa

Pearls	Pitfalls
Hydrate the interpositional graft with autologous minced bursal tissue	Excessive graft manipulation can lead to degradation or breakage
Ensure adequate footprint preparation and proper measurement for enthesis graft	Failure to differentiate cortical from soft tissue surface of the biphasic graft
Removal of dam from 12‐mm cannula allows graft passage to the repair site	Use of a small gauge needle can cause difficulty with injection of bursal tissue
Maintaining an extra medial row suture can assist with temporary fixation of the graft to the tuberosity	Suction collection device can get clogged
Prior to securing the lateral row, ensure the graft is interposed between the tendon and footprint	

RCR, rotator cuff repair.

### Postoperative Rehabilitation

Patients follow a standard rehabilitation protocol after arthroscopic RCR. There are no special considerations or modifications in patients treated with augmentation. A postoperative sling is applied in the operating room. Home exercises are initiated immediately after surgery. These include pendulums, as well as elbow, wrist, and hand range of motion (ROM) exercises for the first 2 weeks. From 2 to 6 weeks, the patient works on passive ROM but otherwise remains in the sling. From 6 to 12 weeks, the patient can initiate scapular ROM exercises and begin active‐assisted ROM shoulder exercises with a transition to active ROM as tolerated. At 12 weeks, the patient can initiate strengthening with resisted motions and isometric exercises with the arm at their side and will advance ROM and strengthening after that point. Return to heavy lifting and sports is generally achieved at 6 to 12 months after surgery.

## DISCUSSION

This technical note details the authors’ preferred transosseous equivalent double‐row repair, but other anchors and sutures are satisfactory based on surgeon preference. Current options for RCR augmentation include structural or scaffold grafts and biologic augmentation with platelet‐rich plasma, bone marrow aspirate concentrate, or autologous subacromial bursa. Although differing strategies have been shown to be effective in clinical studies, there is no gold‐standard technique for RCR augmentation. The rotator cuff enthesis is a highly specialized fibrocartilage interface between the tendon and tuberosity, and there is no single strategy to restore the enthesis during RCR.^13^ Therefore, an optimal approach requires consideration of biologic, mechanical, and environmental factors.

Multiple scaffolds have been developed to augment RCR, including xenografts, allografts, and synthetic matrices.^14^ These constructs are intended to facilitate rotator cuff healing through host tissue ingrowth and restoration of a functional tendon‐bone interface. Preclinical investigations of inlay scaffold augmentation have been shown to resemble the native enthesis.^8^ The biphasic interpositional allograft is designed to further replicate the graded transition from soft tissue to bone that characterizes the native rotator cuff insertion, with the aim of promoting native tissue ingrowth at the repair site.^9^, ^10^

The subacromial bursa has been evaluated as an autologous biologic augmentation because of its accessibility, frequent removal during RCR, and source of mesenchymal stem cells.^15^, ^16^, ^17^ A technique for the implantation of minced autologous subacromial bursa has been previously described; however, its efficacy when used alone in RCR is not supported in subsequent clinical studies.^3^, ^11^, ^18^ Notably, there were favorable trends in the subacromial augment group with increased supraspinatus tendon thickness and a higher percentage of Sugaya type 1 tendons at 6 months postoperatively.^11^ One concern regarding previously described techniques is the ability to maintain the collected subacromial tissue at the repair site. This technique allows for the graft to be hydrated in autologous subacromial tissue and for direct implantation of the collected tissue into the porous layer of the graft at the repair site.

In summary, this article details a technique for RCR augmentation using an interpositional allograft supplemented with autologous subacromial bursa. Advantages and disadvantages of this technique are outlined in Table 2. Although clinical studies are required to evaluate the results of this technique, it offers a unique combination of biologic and inlay graft augmentation.

**TABLE 2:** Advantages and Disadvantages of Arthroscopic RCR: A Biphasic Interpositional Allograft and Minced Autologous Subacromial Bursa

Advantages	Disadvantages
Combines biologic and graft augmentation	Increased operative time
Uses an interpositional biphasic graft to optimize tendon‐to‐bone healing	Increased patient cost
Uses autologous tissue from a standard subacromial bursectomy	Unproven efficacy
Directed delivery of MSCs to graft and repair site	Graft does not provide structural support
Does not require additional suture or anchors for fixation	Requires use of a suction collection device

MSCs, mesenchymal stem cells; RCR, rotator cuff repair.

## DISCLOSURES

The author (J.M.G.) declares the following financial interests/personal relationships, which may be considered potential competing interests: J.M.G. reports grants or contracts from any entity from Arthrex (Grant No. US20113); consulting fees from Stryker and Arthrex (personal fees); support for attending meetings and/or travel from Stryker; leadership or fiduciary role in other board, society, committee, or advocacy group, paid or unpaid, from American Academy of Orthopaedic Surgeons (Shoulder/Elbow Program Committee) and ASES (Education Committee); and stock or stock options from BioEnthesis. The other authors (M.D.S., S.K.A.) declare that they have no known competing financial interests or personal relationships that could have appeared to influence the work reported in this article.

## References

[1] Davey MS , Hurley ET , Carroll PJ , et al. Arthroscopic rotator cuff repair results in improved clinical outcomes and low revision rates at 10‐year follow‐up: A systematic review. Arthroscopy. 2023;39:452‐458. 10.1016/j.arthro.2022.11.002 36604006

[2] Plachel F , Jo OI , Rüttershoff K , Andronic O , Ernstbrunner L . A systematic review of long‐term clinical and radiological outcomes of arthroscopic and open/mini‐open rotator cuff repairs. Am J Sports Med. 2023;51:1904‐1913. 10.1177/03635465211073332 35179393

[3] Pancholi N , Gregory JM . Biologic augmentation of arthroscopic rotator cuff repair using minced autologous subacromial bursa. Arthrosc Tech. 2020;9:e1519‐e1524. 10.1016/j.eats.2020.06.013 PMC7587230 33134054

[4] Yanke A , Dandu N , Credille K , Damodar D , Wang Z , Cole BJ . Indications and technique: Rotator cuff repair augmentation. J Am Acad Orthop Surg. 2023;31:1205‐1210. 10.5435/JAAOS-D-23-00101 37816190

[5] Dandu N , Knapik DM , Zavras AG , Garrigues GE , Yanke AB . Arthroscopic rotator cuff repair with biphasic interpositional allograft augmentation. Arthrosc Tech. 2022;11:e483‐e489. 10.1016/j.eats.2021.11.021 PMC9051627 35493049

[6] Puckett CD , Brady D , Thiel K , Perry NM , Rogalski BL , Reynolds AW . Subscapularis repair technique with biphasic cancellous allograft in anatomic total shoulder arthroplasty. Arthrosc Tech. 2025;14:103825. 10.1016/j.eats.2025.103825 PMC12598225 41220680

[7] Forsythe B , Gamsarian V , Mirle V , et al. Rotator cuff repair with a novel interpositional allograft augmentation. Video J Sports Med. 2023;3:26350254231159425. 10.1177/26350254231159425 PMC11938094 40309413

[8] Villarreal‐Espinosa JB , Berreta RS , Boden SA , et al. Inlay scaffold augmentation of rotator cuff repairs enhances histologic resemblance to native enthesis in animal studies: A systematic review. Arthroscopy. 2025;41:2048‐2060. 10.1016/j.arthro.2024.06.048 39029812

[9] Credille KT , Wang ZRC , Horner NS , et al. Biphasic interpositional allograft for rotator cuff repair augmentation is safe in an ovine model. Arthroscopy. 2023;39:1983‐1997. 10.1016/j.arthro.2023.03.018 37001743

[10] Dickerson DA , Misk TN , Van Sickle DC , Breur GJ , Nauman EA . In vitro and in vivo evaluation of orthopedic interface repair using a tissue scaffold with a continuous hard tissue‐soft tissue transition. J Orthop Surg. 2013;8:18. 10.1186/1749-799X-8-18 PMC3689086 23782505

[11] Gregory JM , Smith AZ , Zandiyeh P . Effect of subacromial bursa augmentation on self‐reported and magnetic resonance imaging outcomes in arthroscopic rotator cuff repair: A prospective, blinded randomized clinical trial. Am J Sports Med. 2026;54:848‐856. 10.1177/03635465251407328 PMC13359769 41549447

[12] Cole BJ , Kaiser JT , Wagner KR , et al. Prospective randomized trial of biologic augmentation with bone marrow aspirate concentrate in patients undergoing arthroscopic rotator cuff repair. Am J Sports Med. 2023;51:1234‐1242. 10.1177/03635465231154601 36811557

[13] Abdalla AA , Pendegrass CJ . Biological approaches to the repair and regeneration of the rotator cuff tendon‐bone enthesis: A literature review. Biomater Transl. 2023;4:85‐103. 10.12336/biomatertransl.2023.02.004 PMC10817785 38283917

[14] Thangarajah T , Pendegrass CJ , Shahbazi S , Lambert S , Alexander S , Blunn GW . Augmentation of rotator cuff repair with soft tissue scaffolds. Orthop J Sports Med. 2015;3:2325967115587495. 10.1177/2325967115587495 PMC4622366 26665095

[15] Morikawa D , Muench LN , Baldino JB , et al. Comparison of preparation techniques for isolating subacromial bursa‐derived cells as a potential augment for rotator cuff repair. Arthroscopy. 2020;36:80‐85. 10.1016/j.arthro.2019.07.024 31708351

[16] Utsunomiya H , Uchida S , Sekiya I , Sakai A , Moridera K , Nakamura T . Isolation and characterization of human mesenchymal stem cells derived from shoulder tissues involved in rotator cuff tears. Am J Sports Med. 2013;41:657‐668. 10.1177/0363546512473269 23371475

[17] Morikawa D , Johnson JD , Kia C , et al. Examining the potency of subacromial bursal cells as a potential augmentation for rotator cuff healing: An in vitro study. Arthroscopy. 2019;35:2978‐2988. 10.1016/j.arthro.2019.05.024 31629585

[18] Gregory JM , Ybarra C , Liao Z , Kumaravel M , Patel S , Warth RJ . Clinical outcomes of rotator cuff repair with subacromial bursa reimplantation: A retrospective cohort study. JSES Int. 2023;7:763‐767. 10.1016/j.jseint.2023.05.010 PMC10499868 37719828

